# Association of psychological distress, smoking and genetic risk with the incidence of lung cancer: a large prospective population-based cohort study

**DOI:** 10.3389/fonc.2023.1133668

**Published:** 2023-07-13

**Authors:** Jing Zhang, Yi Wang, Tingting Hua, Xiaoxia Wei, Xiangxiang Jiang, Mengmeng Ji, Zhimin Ma, Yanqian Huang, Hui Wang, Lingbin Du, Meng Zhu, Lin Xu, Weibing Wu, Hongxia Ma

**Affiliations:** ^1^ Department of Epidemiology, Center for Global Health, School of Public Health, Nanjing Medical University, Nanjing, China; ^2^ Department of Respiratory Diseases, Nanjing Chest Hospital, Nanjing, China; ^3^ Department of Cancer Prevention, The Cancer Hospital of the University of Chinese Academy of Sciences (Zhejiang Cancer Hospital), Hangzhou, China; ^4^ Institute of Basic Medicine and Cancer (IBMC), Chinese Academy of Sciences, Hangzhou, China; ^5^ Jiangsu Key Lab of Cancer Biomarkers, Prevention and Treatment, Collaborative Innovation Center for Cancer Personalized Medicine, Nanjing Medical University, Nanjing, China; ^6^ Department of Thoracic Surgery, Jiangsu Key Laboratory of Molecular and Translational Cancer Research, Jiangsu Cancer Hospital, Jiangsu Institute of Cancer Research, The Affiliated Cancer Hospital of Nanjing Medical University, Nanjing, China; ^7^ Department of Thoracic Surgery, The First Affiliated Hospital of Nanjing Medical University, Nanjing, China; ^8^ Research Units of Cohort Study on Cardiovascular Diseases and Cancers, Chinese Academy of Medical Sciences, Beijing, China

**Keywords:** lung cancer, psychological distress, smoking, genetic susceptibility, UK Biobank

## Abstract

**Background:**

Emerging evidence suggests a potential link between psychological distress (anxiety and depression) and lung cancer risk, however, it is unclear whether other factors such as tobacco smoking and genetic susceptibility modify the association.

**Methods:**

We included 405,892 UK Biobank participants free of cancer at baseline. Psychological distress was measured using the Patient Health Questionnaire-4 (PHQ-4). A polygenic risk score (PRS) was calculated using 18 lung cancer-associated genetic loci. Multivariable Cox regression models were used to estimate hazard ratios (HRs) and 95% confidence intervals (CIs).

**Results:**

During a median follow-up of 7.13 years, 1754 lung cancer cases were documented. The higher score of psychological distress was associated with an increased risk of lung cancer (HR_per 1-SD_= 1.07, 95% CI: 1.02-1.11) after adjustment for smoking and other confounders. Mediation analysis revealed that 16.8% (95% CI: 13.0%-20.6%) of the distress-lung cancer association was mediated by smoking. Compared with never smokers with no distress, participants with heavy smoking and high distress had the highest risk of lung cancer (HR=18.57, 95% CI: 14.51-23.76). Both multiplicative and additive interactions were observed between smoking and psychological distress in lung cancer. Furthermore, the greatest relative increase in risk was observed among those with high genetic risk and high distress (HR=1.87, 95%CI: 1.50-2.33), and there was a significant additive interaction between the PRS and psychological distress.

**Conclusion:**

Our results indicate that psychological distress was associated with an elevated risk of incident lung cancer, and such relation was modified by tobacco smoking and genetic susceptibility.

## Introduction

Lung cancer is the second most common cancer, with an estimated 2.20 million new cancer cases worldwide in 2020, and the leading cause of cancer death (1). Despite improvements being made in diagnostics and treatment strategies in recent decades, the prognosis of lung cancer remains poor, with a 5-year survival of less than 20% (2). Smoking is a well-established risk factor for lung cancer; nevertheless, it is estimated that 10%-15% of all lung cancers are attributed to factors other than tobacco (3, 4). Therefore, it is vital to identify additional modifiable and avoidable risk factors for primary prevention, as well as to identify upstream determinants of smoking.

Psychological distress is generally defined as a state of poor mental health characterized by symptoms of depression and anxiety (5). A series of studies have shown an association between psychological distress and an elevated risk of mortality (6), diabetes (7), cardiovascular disease (8), and cancer (9). Previous studies suggest potential links between psychological distress and lung cancer risk (6, 10–16); however, most of these studies were of relatively small size and reported inconsistent results. In addition, compelling evidence has shown that psychological distress is related to behavioral risk factors of lung cancer (17, 18), notably cigarette smoking. For instance, higher levels of psychological distress are associated with an individual’s subsequent smoking habits (18), which further increases the risk of lung cancer. Nevertheless, it is still unclear whether smoking mediates or modifies the association between psychological distress and lung cancer risk.

Additionally, it has been also established that both genetic and behavioral factors may contribute to the development of lung cancer (19, 20). In recent years, emerging evidence has revealed that genetic factors may modify the environment-diseases relation. For example, the previous study has indicated that the association between air pollution exposure and lung cancer could be modified by genetic susceptibility (21). However, investigations on the modification effect of genetic susceptibility on the association between psychological distress and lung cancer risk are scarce. Therefore, the interaction or joint relation between genetic susceptibility and psychological distress in the development of lung cancer still deserves further exploration, which may provide greater insight into lung cancer etiology and prevention strategies.

In this study, we prospectively examined the association between psychological distress and the risk of incident lung cancer based on the UK Biobank cohort, and particularly examined the potential modifying effect of smoking and genetic susceptibility on the association. Specifically, we performed a mediation analysis to assess whether smoking mediated the distress-lung cancer association, and further assessed the joint or interaction effects of smoking and genetic susceptibility with psychological distress in lung cancer risk.

## Subjects and methods

### Study design and participants

The detailed study design and methods of the UK Biobank have been described elsewhere (22). Briefly, UK Biobank is a large-scale prospective population-based cohort study with over 500,000 volunteers aged 40-69 years recruited in 2006-2010. The information on social demographics, lifestyle and other health-related information was collected through touch-screen questionnaires and physical measurements. Blood samples were collected for genotyping. The UK Biobank has approval from the North West Multi-center Research Ethics Committee. All participants provided written informed consent for the study.

Among the 502,507 participants with available data, we excluded participants with prevalent cancer at recruitment (n=46,533), self-reported gender differed from genetic sex (n=318), missing data on smoking status (n=2,666) and psychological distress (n =47,098), leaving a total of 405,892 participants in the primary analysis. In addition, 394,061 individuals with available genetic information were included in the further genetic analysis. The details of the process for the construction of the analytical cohort are shown in **Supplementary Figure 1**.

### Exposure ascertainment

Psychological distress was measured using the 4-item Patient Health Questionnaire (PHQ-4) (23), which is a brief self-report questionnaire consisting of a 2-item depression scale (PHQ-2) and a 2-item anxiety scale (GAD-2) (24). Responses to each item were either “not at all” (scored 0), “several days” (scored 1), “more than half of the days” (scored 2), or “nearly every day” (scored 3). Therefore, the total score ranges from 0 to 12, with a higher score indicating greater distress. To determine a possible dose-response relationship, participants were divided into four groups based on quartiles of the PHQ-4 score: 0, 1, 2-3, and 4-12.

### Assessment of smoking and other covariates

Covariates were selected based on scientific plausibility and prior evidence (10, 25). According to the smoking status from the respondents’ self-report, participants were classified into never, former, or current smokers. Pack-years smoking (PY) was calculated based on self-reported information on age at smoking initiation, the number of cigarettes smoked daily, age of smoking cessation (for former smokers) and age at recruitment (for current smokers). Subsequently, we categorized smoking levels as non-smoking (never smokers), light smoking (PY<30), and heavy smoking (PY≥30).

Other covariate data were collected at baseline using standard protocols, including socioeconomic characteristics (age at recruitment, sex, ethnic background, education, Townsend deprivation index and family history of lung cancer), and lifestyle factors (healthy diet score, BMI and physical activity). The healthy diet score was calculated based on the following diet factors: fruits: ≥3 pieces/day; vegetables: ≥ 4 tablespoons/day; fish: ≥ 2 times/week; unprocessed red meat intake ≤ 2 times/week; and processed meat intake ≤ 2 times/week (26). Missing data on covariates were coded as a missing indicator for categorical variables and with sex-specific median values for continuous variables.

### Polygenic risk score calculation

Detailed information on the procedure for genotyping, imputation and quality control in the UK Biobank cohort has been previously reported (27). In the present study, we created a polygenic risk score (PRS) for lung cancer using 18 independent single nucleotide polymorphisms (SNPs) based on the largest available lung cancer genome-wide association studies (GWAS) of European descent (**Supplementary Table 1**) (28). The PRS was calculated using the equation: PRS =β1 × SNP1 + β2 × SNP2 +…+βn × SNPn. Individual SNP was recoded as 0, 1, or 2 according to the number of risk alleles, and the effect size (β-coefficient) for each SNP was obtained from the GWAS data. According to PRS, we classified participants into three groups of low (lowest tertile), intermediate (second tertile) and high (highest tertile) genetic risk of lung cancer.

### Outcome ascertainment

In UK Biobank study, cancer cases were identified through linkage to national cancer registries in England, Wales, and Scotland. The complete follow-up date was March 31, 2016 for England and Wales, and October 31, 2015 for Scotland. We defined lung cancer outcome according to the 10th Revision of the International Classification of Diseases (ICD-10): C33 and C34. Participants were followed-up until the date of the diagnosis of lung cancer, death, loss to follow-up, or censoring date (March 31, 2017, for England; October 31, 2016, for Scotland; and February 29, 2016, for Wales), whichever came first.

### Statistical analysis

Cox proportional hazards model was used to estimate the hazard ratio (HR) and corresponding 95% confidence intervals (CIs). Follow-up time was treated as the time scale. The proportional hazards assumption was tested using Schoenfeld residuals. Psychological distress was tested as a categorical variable split into quartiles and as a continuous variable (per 1-standard deviation (19) increment), respectively. The first model was adjusted for age at recruitment (continuous), sex, ethnic background (white, non-white), education (college or university degree, no degree), Townsend deprivation index (quintiles) and family history of lung cancer (no, yes). A second model further adjusted for smoking, including smoking status (never, former, current) and pack-years of smoking (continuous). In the third model, other factors include healthy diet score (continuous), BMI (kg/m2, <25, 25-29.9, ≥30), and physical activity (MET-h/week, <10, 10-50, ≥50) were added to the first model. Lastly, the fourth model contained all the covariates mentioned above. For the genetic analyses, we further adjusted for the first ten genetic principal components and the genotyping array.

Psychological distress was also significantly associated with smoking, the most important risk factor of lung cancer. Thus, psychological distress could probably influence lung cancer risk by increasing smoking amount. To further clarify the causal path of psychological distress on lung cancer risk, the causal mediation analysis was implemented within a Cox proportional hazard framework to assess mediation by pack-years of smoking on the distress-lung cancer association. With these models, we estimated the direct effect of continuous psychological distress and the indirect effect mediated through continuous pack-years. The mediation analysis was performed using the R packages of “regmedint”, based on the product method proposed by Valeri and Vanderweele (29, 30). In addition, we also evaluated whether the association between psychological distress and lung cancer risk differed by smoking level or PRS by using multiplicative and additive interaction analyses. To quantify multiplicative interactions, we added an interaction term in the Cox proportional hazards regression models. In order to evaluate the interaction effect of smoking and psychological distress, we put the multiplication term of smoking and psychological distress in the model as the interaction term. In the model evaluating the interaction effect of PRS and psychological distress, we put the multiplication term of PRS and psychological distress as the interaction term. Relative excess risk due to interaction (RERI) and the attributable proportion because of the interaction (AP) was used to measure the interaction on the additive scale (31). The 95% CIs of the RERI and AP would not include 0 if there was additive interaction (32).

To assess the robustness of the results, we conducted several sensitivity analyses: (1) excluding participants who with less than two years of follow-up; (2) reclassified smoking levels based on 20 pack-years of smoking (none: never smoker, light: PY<20, and heavy: PY ≥20) (33); (3) genetic analysis only included participants of European descent. All analyses were performed using R Software (version 3.6.0), and a two-sided P-value <0.05 was considered to be statistically significant.

## Results

### Population characteristics

During a median of follow-up time 7.1 years (IQR 6.4-7.7 years), 1754 incident lung cancer cases were recorded. **Table 1** reports the baseline characteristics of the participants according to psychological distress categories. Participants with higher levels of psychological distress were more likely to be slightly younger, female, less educated, and more deprived. In addition, they were more likely to have adverse lifestyle factors (smoking, obesity, physical inactivity and unhealthy diet).

**Table 1:** Baseline characteristics of UK biobank participants according to psychological distress categories.

Characteristic	Quartile categories of psychological distress			
	Quartile 1	Quartile 2	Quartile 3	Quartile 4
Participants (No.)	161087	97984	83843	62978
Age at baseline (years), mean (SD)	57.63 (7.81)	56.13 (8.11)	55.1 (8.16)	54.09 (7.94)
Female, n (%)	76741 (47.64)	54841 (55.97)	46721 (55.72)	36475 (57.92)
White ethnic background, n (%)	154278 (95.77)	94483 (96.43)	79177 (94.43)	57161 (90.76)
College or university degree, n (%)	54599 (33.89)	35415 (36.14)	29791 (35.53)	17424 (27.67)
Townsend deprivation index, mean (SD)	-1.70 (2.87)	-1.55 (2.91)	-1.24 (3.09)	-0.45 (3.42)
Family history of lung cancer, n (%)	19120 (11.87)	11758 (12.00)	10160 (12.12)	8378 (13.30)
Smoking status, n (%)				
Never smoker	91867 (57.03)	54685 (55.81)	45463 (54.22)	31667 (50.28)
Former smoker	56281 (34.94)	34311 (35.02)	29069 (34.67)	20227 (32.12)
Current smoker	12939 (8.03)	8988 (9.17)	9311 (11.11)	11084 (17.60)
Pack-years of smoking, mean (SD)	8.87 (13.86)	9.14 (14.25)	9.82 (15.00)	12.08 (17.67)
BMI (kg/m^2^), mean (SD)				
Normal (<25 kg/m^2^)	55409 (34.40)	32607 (33.28)	27681 (33.02)	18324 (29.10)
Overweight (25-<30 kg/m^2^)	72921 (45.27)	41648 (42.50)	35035 (41.79)	24819 (39.41)
Obese (≥30 kg/m^2^)	32757 (20.33)	23729 (24.22)	21127 (25.20)	19835 (31.50)
Physical activity, n (%)				
Low (<10 MET-h/week)	19667 (12.21)	15305 (15.62)	14113 (16.83)	13000 (20.64)
Moderate (10-<50 MET-h/week)	94733 (58.81)	59099 (60.31)	50025 (59.67)	36613 (58.14)
High (≥50 MET-h/week)	46687 (28.98)	23580 (24.07)	19705 (23.50)	13365 (21.22)
Healthy diet score, n (%)				
0-1	17190 (10.67)	11485 (11.72)	10810 (12.89)	9655 (15.33)
2-3	76149 (47.27)	48188 (49.18)	41689 (49.72)	32362 (51.39)
4-5	67748 (42.06)	38311 (39.10)	31344 (37.38)	20961 (33.28)

BMI, body mass index; MET, metabolic equivalent.

### Association between psychological distress and risk of incident lung cancer

The higher score of psychological distress was associated with an increased risk of incident lung cancer in a dose-response fashion (**Table 2** and **Supplementary Figure 2**). In primary models, psychological distress was significantly associated with a higher risk of incident lung cancer (HR_per 1-SD_= 1.21, 95% CI: 1.16-1.26). However, after further adjustment for smoking status and pack-years of smoking, the relationship was substantially attenuated (HR_per 1-SD_= 1.07, 95% CI: 1.03-1.12), and this association did not appreciably alter after further adjustment for other lifestyle factors (HR_per 1-SD_= 1.07, 95% CI: 1.02-1.11) (**Table 2**). These results suggested that smoking might be an important mediator of the distress-lung cancer association. In the sensitivity analyses, results did not change appreciably after excluding individuals with less than two years of follow-up (**Supplementary Table 2**).

**Table 2:** Associations between psychological distress and the risk of lung cancer.

	No. cases/Person years	Model 1: Sociodemographic factors ^a^			Model 2: Model 1 + smoking ^b^		Model 3: Model 1 + other factors ^c^		Model 4: All covariates ^d^	
		HR (95%CI)	*P* value		HR (95%CI)	*P* value	HR (95%CI)	*P* value	HR (95%CI)	*P* value
Distress categories										
Quartile 1	622/1139612	1.00 (ref)			1.00 (ref)		1.00 (ref)		1.00 (ref)	
Quartile 2	397/693101	1.20 (1.06-1.36)	4.28×10^-3^		1.10 (0.97-1.25)	0.139	1.18 (1.04-1.34)	0.010	1.10 (0.97-1.24)	0.158
Quartile 3	368/593621	1.38 (1.21-1.57)	1.48×10^-6^		1.19 (1.05-1.36)	7.81×10^-3^	1.34 (1.17-1.52)	1.28×10^-5^	1.18 (1.04-1.35)	0.011
Quartile 4	367/443704	1.85 (1.62-2.11)	8.59×10^-20^		1.27 (1.11-1.45)	6.33×10^-4^	1.76 (1.54-2.01)	1.19×10^-16^	1.25 (1.09-1.43)	1.35×10^-3^
***P* value for trend**		1.13×10^-19^			2.06×10^-4^		1.61×10^-16^		4.78×10^-4^	
**Per 1 SD increment ** ^**e**^		1.21 (1.16-1.26)	2.02×10^-19^		1.07 (1.03-1.12)	1.26×10^-3^	1.19 (1.14-1.24)	6.80×10^-16^	1.07 (1.02-1.11)	2.95×10^-3^

Defined: HR, hazards ratio; CI, confidence interval.

a Model 1: adjusted for age at recruitment, sex, ethnic background, education, Townsend deprivation index, and family history of lung cancer.

b Model 2: model1+ smoking status, and pack-years of smoking.

c Model 3: model1+ healthy diet score, BMI, and physical activity.

d Model 4: all covariates mentioned above.

e SD was the standard deviation of scores, which was 2.11.

Similar positive associations were observed in the stratified analyses according to age at recruitment, sex, ethnic background, education, Townsend deprivation index, family history of lung cancer, smoking status, alcohol intake frequency, BMI, physical activity, healthy diet score, and histological subtypes (all *P*_heterogeneity_>0.05) (**Supplementary Table 3**). Of the individual psychological distress items, depressed mood (HR=1.08, 95% CI: 1.00-1.15), and tiredness/lethargy (HR=1.10, 95% CI: 1.04-1.16) were positively associated with incident lung cancer (**Supplementary Table 4**).

### Mediation analysis of smoking on association between psychological distress and incident lung cancer risk

**Figure 1:**
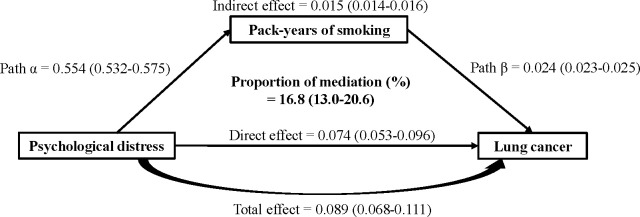
Mediating effects of smoking on the association between psychological distress and lung cancer. Coefficients and 95% confidence intervals are presented: “Path α” is the linear regression coefficient of the distress-smoking association, and “Path β” is the cox proportional hazards regression coefficient of the smoking-lung cancer association. Adjusted confounding factors were age at recruitment, sex, ethnic background, education, Townsend deprivation index, family history of lung cancer, healthy diet score, BMI, and physical activity.

The result of the mediation analysis was shown in **Figure 1** and **Supplementary Table 5**. Mediation analysis further confirmed that the association between psychological distress and risk of lung cancer was partly mediated by smoking. Specifically, participants with higher psychological distress were associated with increased smoking (beta=0.55, 95% CI: 0.53-0.58), and there was a significant direct effect of psychological distress on lung risk (HR=1.08, 95% CI: 1.05-1.10). The indirect effect of smoking was also significant (HR=1.02, 95% CI: 1.01-1.02). These findings indicated that 16.8% (95% CI: 13.0%-20.6%) of the total effect of psychological distress on lung cancer risk was mediated by smoking.

### Joint effect and interaction of smoking or PRS and psychological distress on incident lung cancer risk

**Figure 2:**
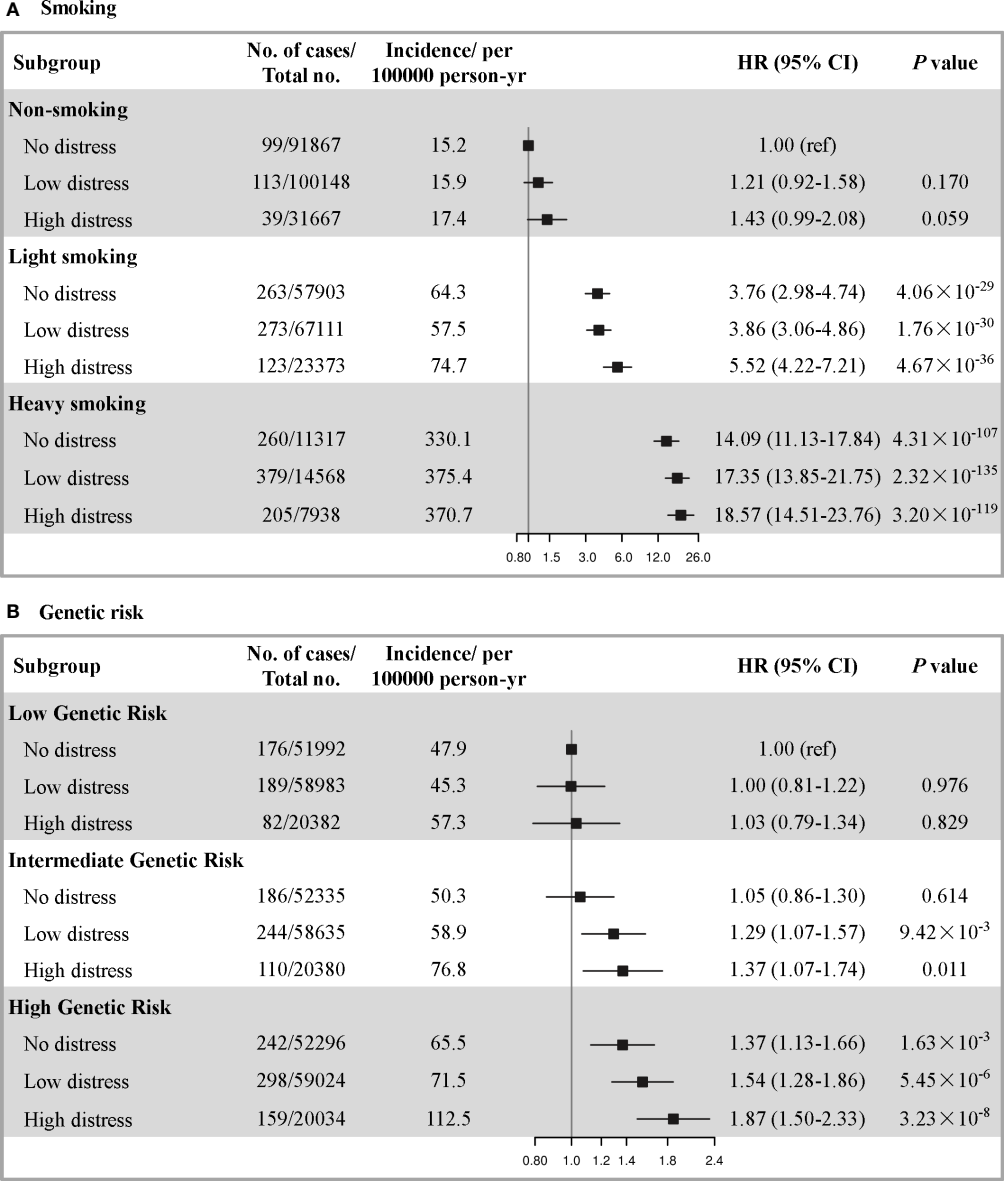
The joint association of **(A)** smoking, **(B)** genetic risk and psychological distress with risk of incident lung cancer. The smoking levels were defined as none (never smoker), light (PY <30) and heavy (PY ≥30). The overall genetic risk was defined as low (lowest tertile), intermediate (second tertile) and high (highest tertile). The psychological distress was defined as none (quartile 1), low (quartiles 2-3) and high (quartile 4). For the smoking, the hazard ratios were estimated using Cox proportional-hazard models with adjustment for age at recruitment, sex, ethnic background, education, Townsend deprivation index, family history of lung cancer, healthy diet score, BMI, and physical activity. For the PRS, another adjusted for smoking status, pack-years of smoking, the first ten principal components of ancestry and genotyping batch.

We found that PRS of lung cancer was significantly associated with an increased risk of incident lung cancer (HR_per 1-SD_= 1.20, 95% CI: 1.15-1.26), which did not change with additional adjustment for psychological distress (**Supplementary Table 6** and **Supplementary Figure 3**). We further observed the joint association of the smoking or PRS with psychological distress on the risk of incident lung cancer in a dose-response manner (*P* trend=3.00×10^-306^ for smoking; *P* trend=2.16×10^-14^ for PRS). Compared with never smokers with no distress, those with heavy smoking and high distress had the highest risk of lung cancer (HR=18.57, 95% CI: 14.51-23.76) (**Figure 2A**). A similar pattern of joint effect was observed for PRS and psychological distress, the greatest relative increase of risk was observed among those with high genetic risk and high distress (HR=1.87, 95%CI: 1.50-2.33) (**Figure 2B**). Additionally, the positive associations between psychological distress and the risk of lung cancer were also observed in the stratified analyses according to smoking levels or PRS categories (**Supplementary Table 7-8**). We repeated the analyses by the reclassification of smoking levels or the inclusion of only participants with European ancestry in genetic analysis, and the results were not materially changed (**Supplementary Figure 4 A, B**).

**Table 3** shows the results of the interaction analysis. We observed both multiplicative (*P*=2.90×10^-8^) and additive interactions between smoking and psychological distress in lung cancer. Specifically, for heavy smokers with high distress, the RERI was 4.05 (95%CI: 0.83- 7.26), which suggested that there would be 4.05 relative excess risk because of the additive interaction, accounting for 22% (95%CI: 7%-36%) of lung cancer risk in participants who had both heavy smoking and high distress. Additionally, there was an additive interaction but not multiplicative (*P*=0.269) interaction of PRS with psychological distress. For participants with high PRS and high distress, RERI was 0.47 (95%CI: 0.05-0.89), and 25% (95%CI: 4%-46%) of the risk of lung cancer exposed to both risk factors was attributable to the additive interaction. The results remained similar after reclassifying smoking levels and excluding participants of non-European descent in genetic analyses (**Supplementary Table 9**).

**Table 3:** Interaction between smoking levels or PRS categories and psychological distress on the risk of incident lung cancer.

	Additive interaction ^a^				Multiplicative interaction ^a^
	Low distress ^b^		High distress ^b^		
	RERI (95% CI)	AP (95% CI)	RERI (95% CI)	AP (95% CI)	*P*-value
**Smoking** ^**c**^					2.90×10^-8^
Light	-0.11 (-0.83-0.61)	-0.03 (-0.21-0.16)	1.33 (0.15-2.50)	0.24 (0.06-0.42)	
Heavy	3.05 (0.55-5.56)	0.18 (0.05-0.31)	4.05 (0.83-7.26)	0.22 (0.07-0.36)	
**PRS** ^**d**^					0.269
Intermediate	0.24 (-0.04-0.53)	0.18 (-0.03-0.41)	0.28 (-0.13-0.49)	0.21 (-0.06-0.48)	
High	0.18 (-0.13-0.49)	0.12 (-0.12-0.32)	0.47 (0.05-0.89)	0.25 (0.04-0.46)	

Define: RERI, relative excess risk due to interaction; AP, attributable proportion due to interaction; CI, confidence interval; PRS, polygenic risk score.

a For the smoking, adjusted for age at recruitment, sex, ethnic background, education, Townsend deprivation index, family history of lung cancer, healthy diet score, BMI, and physical activity. For the PRS, another adjusted for smoking status, pack-years of smoking, the first ten principal components of ancestry and genotyping batch.

b Defined by psychological distress: none (quartile 1), low (quartiles 2-3) and high (quartile 4).

c Defined by smoking levels: none (never smoker), light (PY <30) and heavy (PY ≥30); the non-smoking and no distress group was the reference categories.

d Defined by PRS: low (lowest tertile), intermediate (second tertile) and high (highest tertile); the low PRS and the no distress group was the reference categories.

## Discussion

In this large prospective cohort study, we observed that psychological distress was associated with a higher risk of lung cancer, and smoking was not only a mediator but also had a multiplicative and additive interaction with psychological distress in the development of lung cancer. In addition, there was an additive interaction between PRS and psychological distress in lung cancer.

Previous studies have indicated a link between psychological distress and lung cancer risk. A recent meta-analysis of eight prospective cohort studies reported a positive association between psychological distress and risk of lung cancer, yet statistically significant heterogeneity across studies was detected (34). With a relatively large sample size and comprehensive adjustment of confounding factors, we further confirmed the association between psychological distress and incident lung cancer risk. Several underlying biological pathways may explain the relation. Psychological distress could lead to dysfunctional activation of the autonomic nervous system and the hypothalamic-pituitary-adrenal (HPA) axis, which in turn influence endocrine and immune processes (35). The association between elevated levels of inflammatory markers (such as C-reactive protein, interleukin-1 and interleukin-6) and psychological distress have been well documented (36, 37), and these markers are linked with an increased risk of lung cancer (38, 39). Besides, psychological stress has been found to suppress the activity of DNA repair enzymes and natural killer (NK) cells function (40), which may play pivotal roles in the cancer defense process. However, the exact underlying mechanisms linking psychological distress to lung cancer still need to be elucidated by further functional research.

In addition to the above-mentioned associations, our study extends previous work in several ways. First, psychological distress could lead to alterations in behaviors, such as smoking, which may partly explain the association. So, we thoroughly investigated the potential roles of smoking on the distress-lung cancer association and found that smoking played both a mediating role and an interaction effect in the association between psychological distress and lung cancer risk. These findings were consistent with a previous study, which reported that smoking habits accounted for 38% of the association between depressive symptoms and lung cancer incidence (10). Psychological distress is a modifiable risk factor that promotes smoking initiation and interferes with smoking cessation (41, 42), hence, it should be prioritized as an upstream contributor to smoking behavior. Fortunately, recent evidences have showed that getting physical activity and keeping good sleep can relieve stress effectively (43).

Second, to the best of our knowledge, the current study is the first to examine the joint association and interaction of psychological distress and genetic susceptibility with the risk of incident lung cancer. Our findings showed that the positive association of psychological distress with the risk of lung cancer tended to be stronger in participants with higher genetic risk, as well as a significant additive interaction between genetic risk and psychological distress was observed. These results further support the opinion that the development of lung cancer is the result of the interplay between genetic and environmental risk factors, suggesting that individuals at high genetic risk of lung cancer should pay more attention to their mental health.

Several strengths of this study included the large sample size and long length of follow-up, which provides sufficient power to detect potential associations. In addition, the occurrence and development of lung cancer is a complex network, and it is difficult to precisely or effectively assess the true effect if only a single factor was considered. Therefore, we thoroughly considered the potential roles of smoking to disentangle its confounding, mediating, and modifying effects on the distress-lung cancer association. Besides, we also considered the joint and interactive effect of genetic susceptibility on the association between psychological distress and lung cancer risk. To assess the robustness of the results, we also performed a series of sensitivity analyses to assess the robustness of our findings.

Nevertheless, we also acknowledged several limitations in this study. First, psychological distress was measured only once at baseline, which was not able to take into account the changes in the distress during the follow-up. Second, as psychological distress was self-reported, measurement errors were inevitable. Third, although we controlled for a series of potential confounders, the possibility of residual confounding from unknown or unmeasured confounding factors still exists. Finally, this cohort included participants who were mostly of European descent; therefore, the generalization of the results to other populations should be interpreted with caution.

In conclusion, this large prospective cohort study demonstrated that psychological distress is associated with an elevated risk of incident lung cancer, which was modestly mediated by smoking. Moreover, the interaction of smoking-distress and genetic-distress play important roles in the occurrence and development of lung cancer, which reinforce the importance of multi-factor intervention in the prevention of lung cancer. Further studies are needed to confirm our findings.

## Data availability statement

The datasets presented in this study can be found in online repositories. The names of the repository/repositories and accession number(s) can be found in the article/**Supplementary Material**.

## Ethics statement

The studies involving human participants were reviewed and approved by REC reference: 21/NW/0157. The patients/participants provided their written informed consent to participate in this study.

## Author contributions

HM, WW and JZ conceived and designed the study. JZ, YW and TH conducted the statistical analysis and drafted the initial manuscript. XW, XJ, MJ, ZM, YH, and HW check the accuracy of data and results. LD, MZ and LX critically revised the manuscript. All authors contributed to the article and approved the submitted version.
