# Supplementary material for: Association of psychological distress, smoking and genetic risk with the incidence of lung cancer: a large prospective population-based cohort study
Source: Front Oncol. 2023 Jul 13;13:1133668. doi: 10.3389/fonc.2023.1133668 (PMC10374306; doi:10.3389/fonc.2023.1133668)
Supplement: Supplementary file 1 [file DataSheet_1.docx]

**Supplementary materials**

**List of contents**

**Supplementary Table 1.** Eighteen genome-wide significant (*P* < 5×10^-8^) SNPs associated with lung cancer in the study of McKay et al. (2017)

**Supplementary Table 2.** Associations between psychological distress and the risk of lung cancer after excluding participants with less than 2 years follow-up

**Supplementary Table 3.** Subgroup analyses of the association between psychological distress and the risk of lung cancer

**Supplementary Table 4.** Associations between psychological distress items and the risk of lung cancer

**Supplementary Table 5.** Mediation by pack-years of smoking in the association of psychological distress with incidence lung cancer

**Supplementary Table 6.** Associations between polygenic risk score of lung cancer and the risk of lung cancer in the UK Biobank

**Supplementary Table 7.** Associations between psychological distress and the risk of lung cancer stratified by smoking levels in the UK Biobank

**Supplementary Table 8.** Associations between psychological distress and the risk of lung cancer stratified by PRS categories in the UK Biobank

**Supplementary Table 9.** Sensitivity analyses of the interaction between smoking levels or PRS categories and psychological distress on the risk of incident lung cancer

**Supplementary Figure 1.** Study design and workflow

**Supplementary Figure 2.** The relationship between psychological distress score and incident lung cancer risk in the UK biobank

**Supplementary Figure 3.** The distribution and relationship of polygenic risk score (PRS) with incident lung cancer in UK Biobank

**Supplementary Figure 4.** Sensitivity analyses of incident lung cancer risk according to (A) smoking, (B) genetic risk and psychological distress categories in the UK Biobank

| **Supplementary Table 1. Eighteen genome-wide significant (*P* < 5×10^-8^) SNPs associated with lung cancer in the study of McKay et al. (2017)** | | | | | | | | |
| --- | --- | --- | --- | --- | --- | --- | --- | --- |
| **CHR** | **SNP** | **Position (hg19/b37)** | **EA** | **OA** | **EAF** | **BETA** | **SE** | ***P* value** |
| 1 | rs71658797 | 77967507 | A | T | 0.103 | 0.128 | 0.019 | 3.25×10^-11^ |
| 3 | rs13080835 | 189357199 | T | G | 0.493 | -0.111 | 0.016 | 7.45×10^-12^ |
| 5 | rs7705526 | 1285974 | A | C | 0.342 | 0.222 | 0.018 | 3.80×10^-35^ |
| 6 | rs6920364 | 167377165 | C | G | 0.456 | 0.068 | 0.012 | 1.29×10^-8^ |
| 6 | rs116822326 | 31434111 | G | A | 0.155 | 0.223 | 0.025 | 3.83×10^-19^ |
| 8 | rs11780471 | 27344719 | A | G | 0.06 | -0.141 | 0.025 | 1.69×10^-8^ |
| 8 | rs4236709 | 32410110 | G | A | 0.218 | 0.124 | 0.019 | 1.28×10^-10^ |
| 9 | rs885518 | 21830157 | G | A | 0.101 | 0.155 | 0.025 | 9.96×10^-10^ |
| 10 | rs11591710 | 105687632 | C | A | 0.137 | 0.151 | 0.023 | 6.30×10^-11^ |
| 11 | rs1056562 | 118125625 | T | C | 0.473 | 0.102 | 0.016 | 2.76×10^-10^ |
| 12 | rs7953330 | 998819 | C | G | 0.315 | -0.146 | 0.02 | 7.26×10^-13^ |
| 13 | rs11571833 | 32972626 | T | A | 0.011 | 0.472 | 0.058 | 6.12×10^-16^ |
| 15 | rs55781567 | 78857986 | G | C | 0.367 | 0.26 | 0.012 | 3.08×10^-103^ |
| 15 | rs66759488 | 47577451 | A | G | 0.362 | 0.068 | 0.012 | 2.83×10^-8^ |
| 15 | rs77468143 | 49376624 | G | T | 0.253 | -0.155 | 0.019 | 1.69×10^-16^ |
| 19 | rs56113850 | 41353107 | T | C | 0.44 | -0.123 | 0.014 | 5.02×10^-19^ |
| 20 | rs41309931 | 62326579 | T | G | 0.117 | 0.157 | 0.026 | 1.31×10^-9^ |
| 22 | rs17879961 | 29121087 | G | A | 0.005 | -0.892 | 0.124 | 5.70×10^-13^ |
| *Defined:* SNP, single nucleotide polymorphism; CHR, chromosome; EA, effect allele; OA, other allele; EAF, effect allele frequency; SE, standard error. | | | | | | | | |

| **Supplementary Table 2. Associations between psychological distress and the risk of lung cancer after excluding participants with less than 2 years follow-up** | | | | | | | | | | | | | |  |  |  |  |  |  |  |  |  |  |  |
| --- | --- | --- | --- | --- | --- | --- | --- | --- | --- | --- | --- | --- | --- | --- | --- | --- | --- | --- | --- | --- | --- | --- | --- | --- |
|  | **No. cases / Person years** | **Model 1:**  **Sociodemographic factors ^a^** | |  | **Model 2: Model 1 +**  **smoking ^b^** | |  | **Model 3: Model 1 + other factors ^c^** | |  | **Model 4: All covariates ^d^** | |  |  |  |  |  |  |  |  |  |  |  |  |
|  |  | **HR (95%CI)** | ***P* value** |  | **HR (95%CI)** | ***P* value** |  | **HR (95%CI)** | ***P* value** |  | **HR (95%CI)** | ***P* value** |  |  |  |  |  |  |  |  |  |  |  |  |
| **Distress categories** |  |  |  |  |  |  |  |  |  |  |  |  |  |  |  |  |  |  |  |  |  |  |  |  |
| Quartile 1 | 495/1138945 | 1.00 (ref) |  |  | 1.00 (ref) |  |  | 1.00 (ref) |  |  | 1.00 (ref) |  |  |  |  |  |  |  |  |  |  |  |  |  |
| Quartile 2 | 319/692658 | 1.22 (1.06-1.40) | 6.18×10^-3^ |  | 1.11 (0.97-1.28) | 0.134 |  | 1.19 (1.04-1.38) | 0.014 |  | 1.11 (0.96-1.28) | 0.155 |  |  |  |  |  |  |  |  |  |  |  |  |
| Quartile 3 | 298/593206 | 1.40 (1.21-1.62) | 4.76×10^-6^ |  | 1.22 (1.05-1.41) | 7.96×10^-3^ |  | 1.36 (1.18-1.57) | 3.13×10^-5^ |  | 1.21 (1.04-1.40) | 0.011 |  |  |  |  |  |  |  |  |  |  |  |  |
| Quartile 4 | 298/443255 | 1.89 (1.63-2.19) | 2.89×10^-17^ |  | 1.29 (1.11-1.50) | 1.05×10^-3^ |  | 1.80 (1.55-2.08) | 1.31×10^-14^ |  | 1.27 (1.09-1.48) | 1.94×10^-3^ |  |  |  |  |  |  |  |  |  |  |  |  |
| ***P* value for trend** |  | 2.75×10^-17^ |  |  | 3.34×10^-4^ |  |  | 1.33×10^-14^ |  |  | 6.77×10^-4^ |  |  |  |  |  |  |  |  |  |  |  |  |  |
| **Per 1 SD increment ^e^** |  | 1.21 (1.16-1.27) | 1.44×10^-16^ |  | 1.08 (1.03-1.13) | 2.68×10^-3^ |  | 1.19 (1.14-1.25) | 1.23×10^-13^ |  | 1.07 (1.02-1.12) | 5.25×10^-3^ |  |  |  |  |  |  |  |  |  |  |  |  |
| *Defined*: HR, hazards ratio; CI, confidence interval | | | | | | | | | | | | | | |  |  |  |  |  |  |  |  |  |  |
| ^a^ Model 1: adjusted for age at recruitment, sex, ethnic background, education, Townsend deprivation index, and family history of lung cancer. | | | | | | | | | | | | | | | | | | | | | | | |  |
| ^b^ Model 2: model1+ smoking status, and pack-years of smoking | | | | | | | | | | | | | | | |  |  |  |  |  |  |  |  |  |
| ^c^ Model 3: model1+ healthy diet score, BMI, and physical activity | | | | | | | | | | | | | | | |  |  |  |  |  |  |  |  |  |
| ^d^ Model 4: all covariates mentioned above  ^e^ SD was the standard deviation of scores, which was 2.11 | | | | | | | | | | | | | | |  |  |  |  |  |  |  |  |  |  |

| **Supplementary Table 3. Subgroup analyses of the association between psychological distress and the risk of lung cancer** | | | | | |
| --- | --- | --- | --- | --- | --- |
| **Subgroup** | **No. cases / Person years** | **HR per 1-SD increment ^a^** | ***P* value** | ***P* _heterogeneity_** |  |
| **Age (years)** |  |  |  | 0.818 |  |
| < 60 | 479/1693466 | 1.05 (0.97-1.13) | 0.235 |  |  |
| ≥60 | 1275/1176572 | 1.04 (0.99-1.09) | 0.131 |  |  |
| **Sex** |  |  |  | 0.227 |  |
| Women | 788/1523141 | 1.09 (1.02-1.16) | 7.30×10^-3^ |  |  |
| Men | 966/1346898 | 1.03 (0.97-1.10) | 0.289 |  |  |
| **Ethnic background** |  |  |  | 0.246 |  |
| White race | 1711/2728836 | 1.07 (1.03-1.12) | 1.64×10^-3^ |  |  |
| Non-white | 40/132852 | 0.87 (0.61-1.24) | 0.429 |  |  |
| **Education** |  |  |  | 0.129 |  |
| College or university degree | 300/968153 | 0.98 (0.87-1.10) | 0.753 |  |  |
| Non-degree | 1406/1849957 | 1.08 (1.03-1.14) | 1.46×10^-3^ |  |  |
| **Townsend deprivation index** |  |  |  | 0.051 |  |
| Below median | 653/1445734 | 1.00 (0.93-1.08) | 0.983 |  |  |
| Above median | 1101/1424305 | 1.11 (1.04-1.16) | 6.17×10^-4^ |  |  |
| **Family history of lung cancer** |  |  |  | 0.201 |  |
| No | 1384/2520549 | 1.08 (1.03-1.14) | 1.14×10^-3^ |  |  |
| Yes | 370/349489 | 1.01 (0.92-1.11) | 0.827 |  |  |
| **Smoking status** |  |  |  | 0.592 |  |
| Never | 251/1586950 | 1.10 (0.97-1.24) | 0.143 |  |  |
| Previous | 785/985824 | 1.14 (1.07-1.22) | 4.62×10^-5^ |  |  |
| Current | 718/297265 | 1.09 (1.01-1.17) | 0.027 |  |  |
| **BMI (kg/m^2^)** |  |  |  | 0.451 |  |
| < 25 | 570/948501 | 1.06 (0.99-1.13) | 0.112 |  |  |
| 25-30 | 748/1233678 | 1.10 (1.03-1.17) | 3.81×10^-3^ |  |  |
| ≥30 | 436/687860 | 1.02 (0.93-1.13) | 0.646 |  |  |
| **Physical activity** |  |  |  | 0.764 |  |
| Low | 319/438440 | 1.09 (0.99-1.21) | 0.079 |  |  |
| Moderate | 1020/1704697 | 1.07 (1.01-1.13) | 0.017 |  |  |
| High | 415/726901 | 1.04 (0.95-1.14) | 0.403 |  |  |
| **Healthy diet score** |  |  |  | 0.902 |  |
| 0-1 | 330/347018 | 1.09 (0.99-1.20) | 0.095 |  |  |
| 2-3 | 895/1402307 | 1.06 (1.00-1.13) | 0.059 |  |  |
| 4-5 | 529/1120714 | 1.06 (0.98-1.15) | 0.13 |  |  |
| **Histological subtype** |  |  |  | 0.997 |  |
| Adenocarcinoma | 718/2865769 | 1.06 (0.99-1.14) | 0.075 |  |  |
| Squamous cell carcinoma | 354/2864292 | 1.06 (0.96-1.16) | 0.226 |  |  |
| Small cell carcinoma | 187/2863556 | 1.06 (0.94-1.20) | 0.323 |  |  |
| ^a^ HRs and 95% CIs were estimated using Cox proportional-hazard models with adjustment for age at recruitment, sex, ethnic background, education, Townsend deprivation index, family history of lung cancer, smoking status, pack-years of smoking, healthy diet score, BMI, and physical activity where appropriate. | | | | | |

| **Supplementary Table 4. Associations between psychological distress items and the risk of lung cancer** | | | | | | | | |  | | | | | |  | | | |  | | |  | | |  | |  |  | |  | | |  |  |  |  |  |
| --- | --- | --- | --- | --- | --- | --- | --- | --- | --- | --- | --- | --- | --- | --- | --- | --- | --- | --- | --- | --- | --- | --- | --- | --- | --- | --- | --- | --- | --- | --- | --- | --- | --- | --- | --- | --- | --- |
| **Items ^e^** | **Model 1:**  **Sociodemographic factors ^a^** | |  | **Model 2: Model 1 +smoking ^b^** | |  | **Model 3: Model 1 +other factors ^c^** | | |  | | **Model 4: All covariates ^d^** | | | | | | | | |  |  |  |  |  |  |  |  |  |  |  |  |  |  |  |  |  |
|  | **HR (95%CI)** | ***P* value** |  | **HR (95%CI)** | ***P* value** |  | **HR (95%CI)** | ***P* value** | | |  | | **HR (95%CI)** | | | | ***P* value** | | | |  |  |  |  |  |  |  |  |  |  |  |  |  |  |  |  |  |
| **Depression items (PHQ-2)** |  |  |  |  |  |  |  |  | | |  | |  | | | |  | | | |  |  |  |  |  |  |  |  |  |  |  |  |  |  |  |  |  |
| depressed mood | 1.25 (1.17-1.34) | 3.09×10^-11^ |  | 1.08 (1.01-1.16) | 0.020 |  | 1.23 (1.15-1.31) | 2.74×10^-9^ | | |  | | 1.08 (1.00-1.15) | | | | 0.037 | | | |  |  |  |  |  |  |  |  |  |  |  |  |  |  |  |  |  |
| Unenthusiasm/disinterest | 1.26 (1.18-1.34) | 1.11×10^-11^ |  | 1.07 (1.00-1.15) | 0.054 |  | 1.23 (1.15-1.31) | 1.30×10^-9^ | | |  | | 1.06 (0.99-1.14) | | | | 0.082 | | | |  |  |  |  |  |  |  |  |  |  |  |  |  |  |  |  |  |
| PHQ-2 sum score | 1.15 (1.11-1.19) | 6.02×10^-14^ |  | 1.05 (1.01-1.09) | 0.018 |  | 1.14 (1.09-1.18) | 1.97×10^-11^ | | |  | | 1.04 (1.00-1.08) | | | | 0.034 | | | |  |  |  |  |  |  |  |  |  |  |  |  |  |  |  |  |  |
| **Anxiety items (GAD-2)** |  |  |  |  |  |  |  |  | | |  | |  | | | |  | | | |  |  |  |  |  |  |  |  |  |  |  |  |  |  |  |  |  |
| Tenseness/restlessness | 1.22 (1.14-1.31) | 9.05×10^-9^ |  | 1.07 (0.99-1.14) | 0.075 |  | 1.20 (1.12-1.28) | 2.96×10^-7^ | | |  | | 1.06 (0.99-1.14) | | | | 0.114 | | | |  |  |  |  |  |  |  |  |  |  |  |  |  |  |  |  |  |
| Tiredness/lethargy | 1.25 (1.18-1.31) | 7.80×10^-17^ |  | 1.11 (1.05-1.16) | 2.01×10^-4^ |  | 1.22 (1.16-1.29) | 5.27×10^-11^ | | |  | | 1.10 (1.04-1.16) | | | | 3.91×10^-4^ | | | |  |  |  |  |  |  |  |  |  |  |  |  |  |  |  |  |  |
| GAD-2 sum score | 1.16 (1.12-1.20) | 8.31×10^-18^ |  | 1.06 (1.03-1.10) | 6.42×10^-4^ |  | 1.15 (1.11-1.19) | 1.17×10^-11^ | | |  | | 1.06 (1.02-1.10) | | | | 1.42×10^-3^ | | | |  |  |  |  |  |  |  |  |  |  |  |  |  |  |  |  |  |
| *Defined*: HR, hazards ratio; CI, confidence interval | | | | | | | | | | | | | |  | |  | | | |  |  |  |  |  |  |  |  |  |  |  |  |  |  |  |  |  |  |
| ^a^ Model 1: adjusted for age at recruitment, sex, ethnic background, education, Townsend deprivation index, and family history of lung cancer. | | | | | | | | | | | | | | | | | | | | | | | | | | | | | | | | | | |  |  |  |
| ^b^ Model 2: model1+ smoking status, pack-years of smoking | | | | | | | | | | | | | | | | | | | | | | | | | | | | | | | | | | | | | |
| ^c^ Model 3: model1+ healthy diet score, BMI, and physical activity | | | | | | | | | | | | | | | | | | | | | | |  |  | |  | | |  | |  |  | |  |  |  |  |
| ^d^ Model 4: all covariates mentioned above  ^e^ Per 1-unit increment | | | | | | | | | | | | | | | | | |  | | | | |  |  | |  | | |  | |  |  | |  |  |  |  |

| **Supplementary Table 5. Mediation by pack-years of smoking in the association of psychological distress with incidence lung cancer** | | | | | | |  | |
| --- | --- | --- | --- | --- | --- | --- | --- | --- |
|  | **Model 1: Sociodemographic factors ^a^** | |  | **Model 2: Model 1+other factors ^b^** | |  |  |  |
|  | **HR** | **(95% CI)** |  | **HR** | **(95% CI)** |  |  |  |
| Natural direct effect | 1.08 | (1.06-1.10) |  | 1.08 | (1.05-1.10) |  |  |  |
| Natural indirect effect | 1.017 | (1.016-1.018) |  | 1.02 | (1.01-1.02) |  |  |  |
| Total effect | 1.10 | (1.08-1.12) |  | 1.09 | (1.07-1.12) |  |  |  |
| Proportion mediated (%) | 18.3 (14.4-22.3) | |  | 16.8 (13.0-20.6) | |  |  |  |
| ^a^ Model 1: adjusted for age at recruitment, sex, ethnic background, education, Townsend deprivation index, and family history of lung cancer. | | | | | | | |  |
| ^b^ Model 2: model1+ healthy diet score, BMI, and physical activity | | | | | | | |  |

| **Supplementary Table 6.** Associations between polygenic risk score of lung cancer and the risk of lung cancer in the UK Biobank | | | | | | | | |
| --- | --- | --- | --- | --- | --- | --- | --- | --- |
| **Genetic risk** | **No. cases / Person years** | **Model 1^a^** | |  | **Model 2^b^** | | |  |
|  |  | **HR (95%CI)** | ***P* value** |  | **HR (95%CI)** | ***P* value** | |  |
| **Tertiles** |  |  |  |  |  |  | |  |
| Low | 447/927435 | 1.00 (ref) |  |  | 1.00 (ref) |  | |  |
| Intermediate | 540/926957 | 1.21 (1.06-1.37) | 3.24×10^-3^ |  | 1.21 (1.07-1.37) | 3.08×10^-3^ | |  |
| High | 699/927739 | 1.53 (1.35-1.72) | 3.47×10^-12^ |  | 1.53 (1.35-1.72) | 3.39×10^-12^ | |  |
| ***P* value for trend** |  | 1.90×10^-12^ |  |  | 1.87×10^-12^ |  | |  |
| **Per 1-SD increment ^c^** |  | 1.20 (1.15-1.26) | 2.59×10^-14^ |  | 1.20 (1.15-1.26) | 2.46×10^-14^ | |  |
| *Defined:* HR, hazards ratio; CI, confidence interval | | | | | | |  |  |
| ^a^ Model 1: adjusted for age at recruitment, sex, ethnic background, education, Townsend deprivation index, family history of lung cancer, smoking status, pack-years of smoking, healthy diet score, BMI, physical activity, the first ten principal components of ancestry and genotyping batch. | | | | | | |  |  |
| ^b^ Model 2: model1+ psychological distress score | | | | | | |  |  |

^c^ SD was the standard deviation of scores, which was 0.37.

| **Supplementary Table 7. Associations between psychological distress and the risk of lung cancer stratified by smoking levels in the UK Biobank** | | | | |
| --- | --- | --- | --- | --- |
| **Subgroup** | **No. cases / person years** | **HR (95% CI) ^a^** | ***P* value** |  |
| **Non-smoking** |  |  |  |  |
| No distress | 99/652074 | 1.00 (ref) |  |  |
| Low distress | 113/711111 | 1.20 (0.91-1.57) | 0.200 |  |
| High distress | 39/223765 | 1.49 (1.01-2.18) | 0.042 |  |
| **Light smoking** |  |  |  |  |
| No distress | 263/408785 | 1.00 (ref) |  |  |
| Low distress | 273/474640 | 1.05 (0.88-1.24) | 0.605 |  |
| High distress | 123/164646 | 1.49 (1.20-1.86) | 3.82×10^-4^ |  |
| **Heavy smoking** |  |  |  |  |
| No distress | 260/78753 | 1.00 (ref) |  |  |
| Low distress | 379/100972 | 1.21 (1.03-1.42) | 0.020 |  |
| High distress | 205/55293 | 1.26 (1.04-1.53) | 0.016 |  |
| ^a^ HRs and 95% CIs were estimated using Cox proportional-hazard models with adjustment for age at recruitment, sex, ethnic background, education, Townsend deprivation index, family history of lung cancer, healthy diet score, BMI, and physical activity. | | | | |

| **Supplementary Table 8. Associations between psychological distress and the risk of lung cancer stratified by PRS categories in the UK Biobank** | | | | |  |
| --- | --- | --- | --- | --- | --- |
| **Subgroup** | **No. cases/ person years** | **HR (95% CI) ^a^** | ***P* value** |  |  |
| **Low genetic risk** |  |  |  |  |  |
| No distress | 176/367334 | 1.00 (ref) |  |  |  |
| Low distress | 189/416887 | 0.98 (0.79-1.20) | 0.831 |  |  |
| High distress | 82/143214 | 0.98 (0.75-1.29) | 0.902 |  |  |
| **Intermediate genetic risk** |  |  |  |  |  |
| No distress | 186/369497 | 1.00 (ref) |  |  |  |
| Low distress | 244/414241 | 1.23 (1.01-1.49) | 0.035 |  |  |
| High distress | 110/143219 | 1.28 (1.00-1.64) | 0.052 |  |  |
| **High genetic risk** |  |  |  |  |  |
| No distress | 242/369476 | 1.00 (ref) |  |  |  |
| Low distress | 298/416978 | 1.14 (0.96-1.35) | 0.134 |  |  |
| High distress | 159/141286 | 1.44 (1.17-1.78) | 7.23×10^-4^ |  |  |
| ^a^ HRs and 95% CIs were estimated using Cox proportional-hazard models with adjustment for age at recruitment, sex, ethnic background, education, Townsend deprivation index, family history of lung cancer, smoking status, pack-years of smoking, healthy diet score, BMI, physical activity, the first ten principal components of ancestry and genotyping batch. | | | | | |

| **Supplementary Table 9. Sensitivity analyses of the interaction between smoking levels or PRS categories and psychological distress on the risk of incident lung cancer** | | | | | | | |  |
| --- | --- | --- | --- | --- | --- | --- | --- | --- |
|  | **Additive interaction ^a^** | | | | |  | **Multiplicative interaction ^a^** | |
|  | **Low distress ^b^** | |  | **High distress ^b^** | |  |  |  |
|  | **RERI (95% CI)** | **AP (95% CI)** |  | **RERI (95% CI)** | **AP (95% CI)** |  | ***P*-value** | |
| **Smoking ^c^** |  |  |  |  |  |  | 2.90×10^-8^ | |
| Light | -0.29 (-1.01-0.42) | -0.10 (-0.36-0.15) |  | 0.84 (-0.35-2.03) | 0.20 (-0.05-0.45) |  |  | |
| Heavy | 1.97 (0.34-3.60) | 0.15 (0.04-0.27) |  | 3.75 (1.45-6.05) | 0.25 (0.13-0.37) |  |  | |
| **PRS ^d^** |  |  |  |  |  |  | 0.204 | |
| Intermediate | 0.28 (-0.03-0.59) | 0.21 (-0.02-0.44) |  | 0.28 (-0.14-0.7) | 0.21 (-0.09-0.51) |  |  | |
| High | 0.12 (-0.23-0.46) | 0.07 (-0.14-0.29) |  | 0.47 (0.01-0.93) | 0.25 (0.02-0.47) |  |  | |
| *Define*: RERI, relative excess risk due to interaction; AP, attributable proportion due to interaction; CI, confidence interval; PRS, polygenic risk score. | | | | | | | |  |
| ^a^ For the smoking, models were adjusted for age at recruitment, sex, ethnic background, education, Townsend deprivation index, family history of lung cancer, healthy diet score, BMI, and physical activity. For the PRS, additionally adjusted for smoking status, pack-years of smoking, the first ten principal components of ancestry and genotyping batch.  ^b^ Defined by psychological distress: none (quartile 1), low (quartiles 2-3) and high (quartile 4). | | | | | | | |  |
| ^c^ Defined by smoking levels: none (never smoker), light (PY <20) and heavy (PY ≥20); the non-smoking and the no distress group was the reference categories | | | | | | | |  |
| ^d^ Defined by PRS of white British individuals: low (lowest tertile), intermediate (second tertile) and high (highest tertile); the low PRS and the no distress group was the reference categories. | | | | | | | |  |


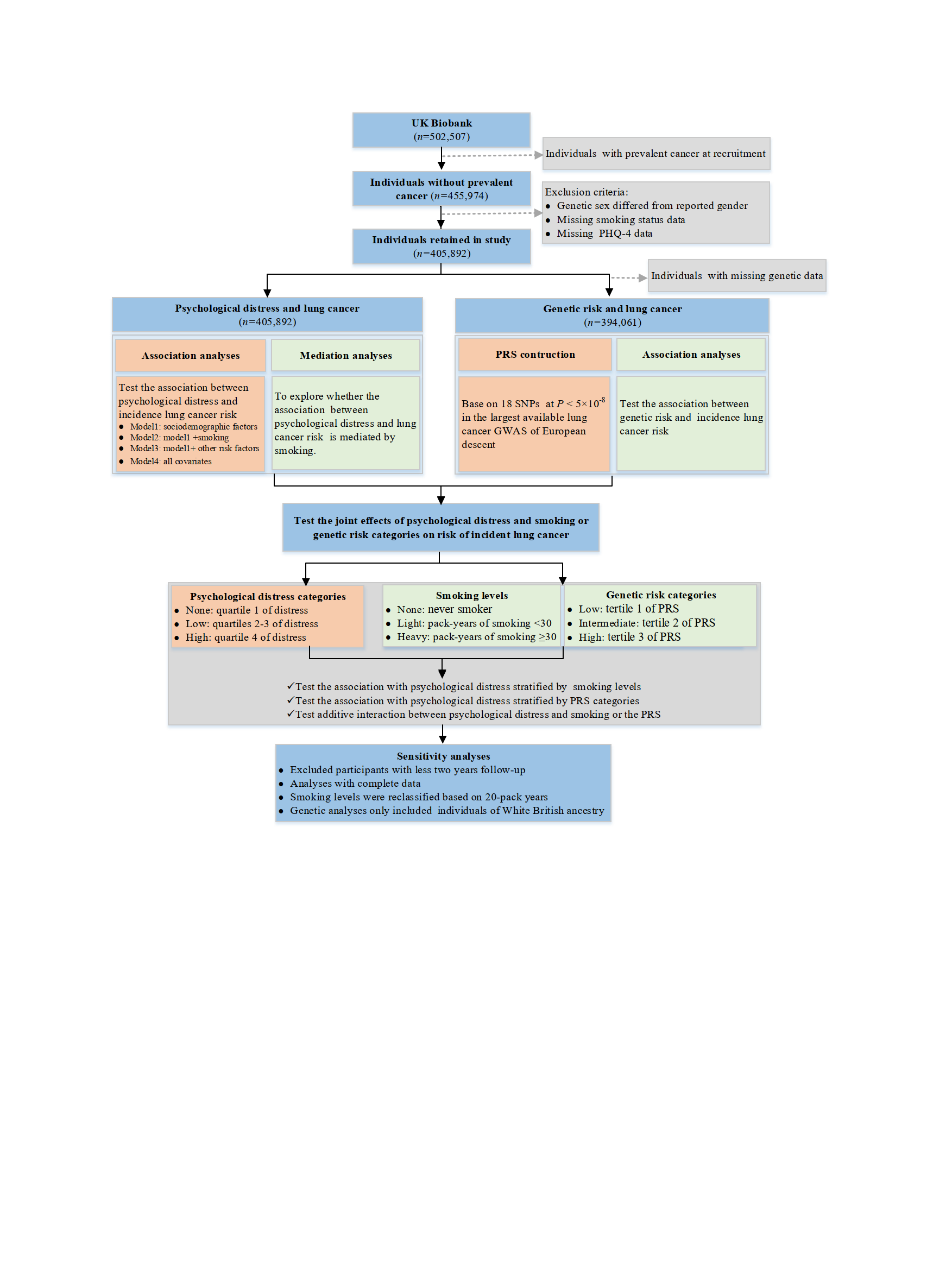


**Supplementary Figure 1. Study design and workflow.**


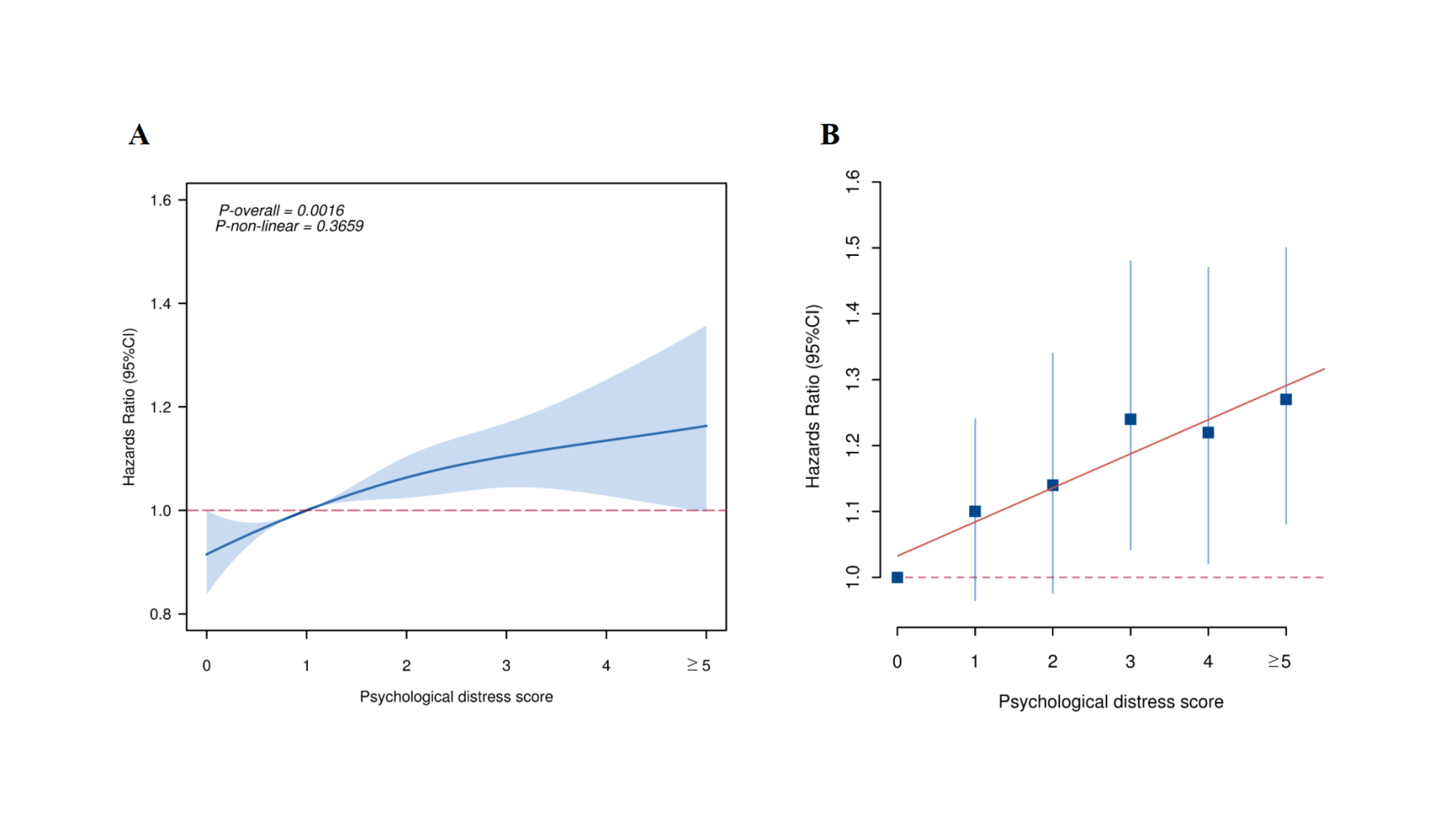


**Supplementary Figure 2. The relationship between psychological distress score and incident lung cancer risk in the UK biobank.** According to the characteristics of psychological distress score distribution, the score greater than 5 was defined as 5. A) Linear relationship between psychological distress score and lung cancer risk was assessed using restricted cubic spline analysis B) Individuals in the UK Biobank were divided into six groups according to their psychological distress score, and HRs for each group were compared with those in no distress (reference group); error bars show the 95%CIs.

Hazard ratios (HRs) were estimated with adjustment for age at recruitment, sex, ethnic background, education, Townsend deprivation index, family history of lung cancer, smoking status, pack-years of smoking, healthy diet score, BMI, and physical activity.

**
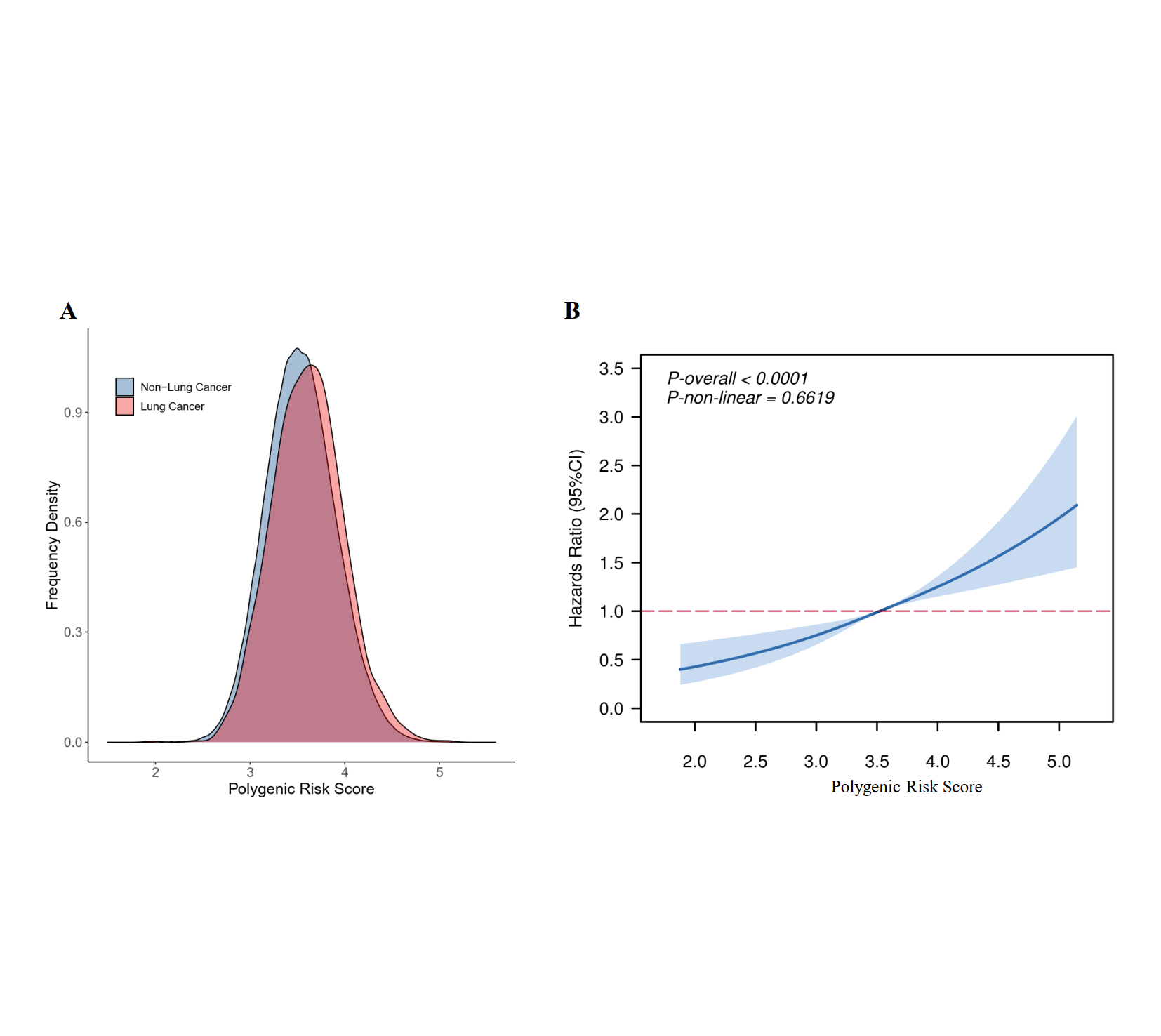
Supplementary Figure 3. The distribution and relationship of polygenic risk score (PRS) with incident lung cancer in UK Biobank.** (A) Distribution of PRS in participants affected with or without lung cancer; (B) Linear relationship between PRS and lung cancer risk was assessed using a restricted cubic spline analysis, and hazard ratios (HRs) were estimated with adjustment for age at recruitment, sex, ethnic background, education, Townsend deprivation index, family history of lung cancer, smoking status, pack-years of smoking, healthy diet score, BMI, physical activity, the first ten principal components of ancestry and genotyping batch.


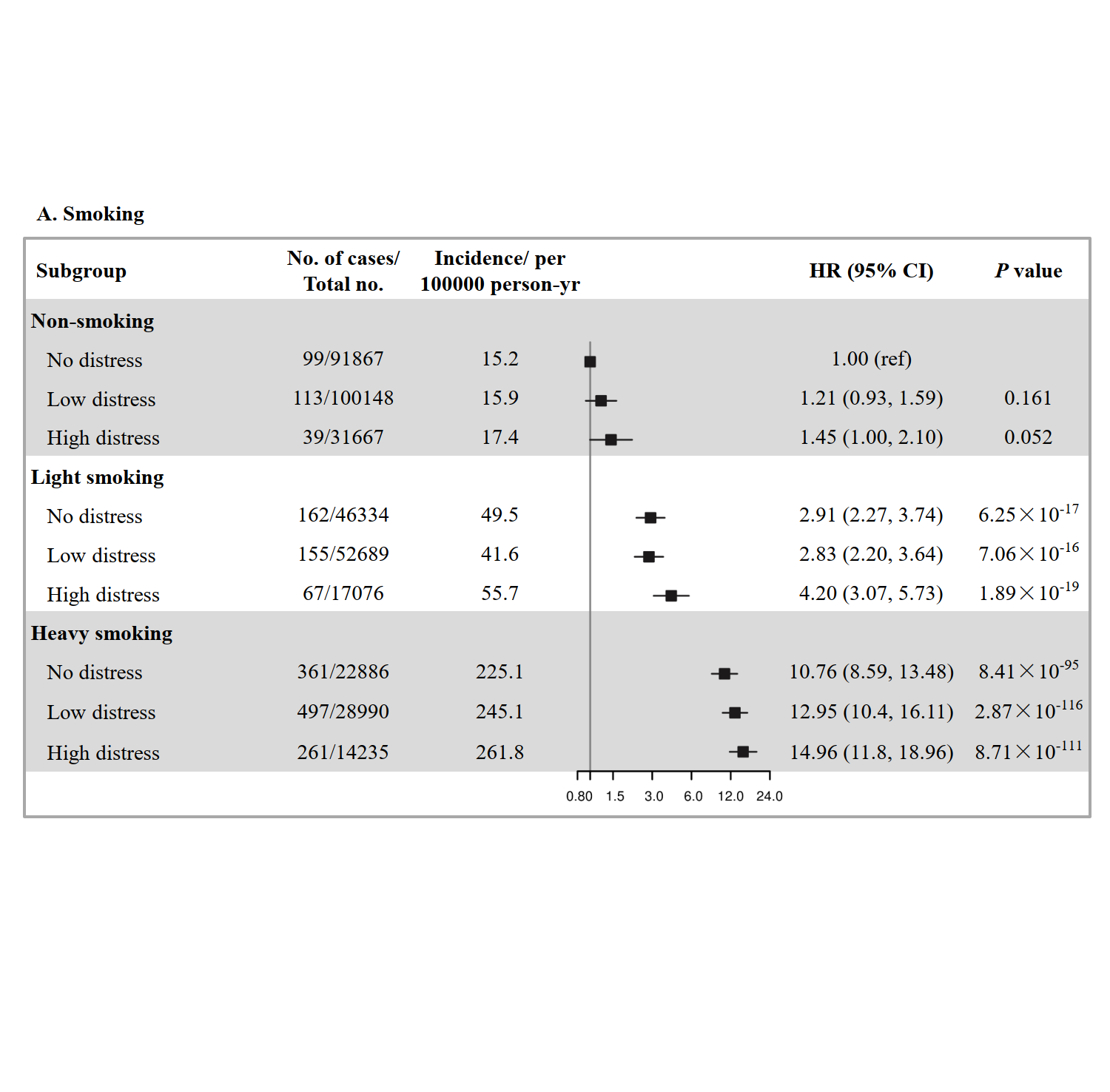

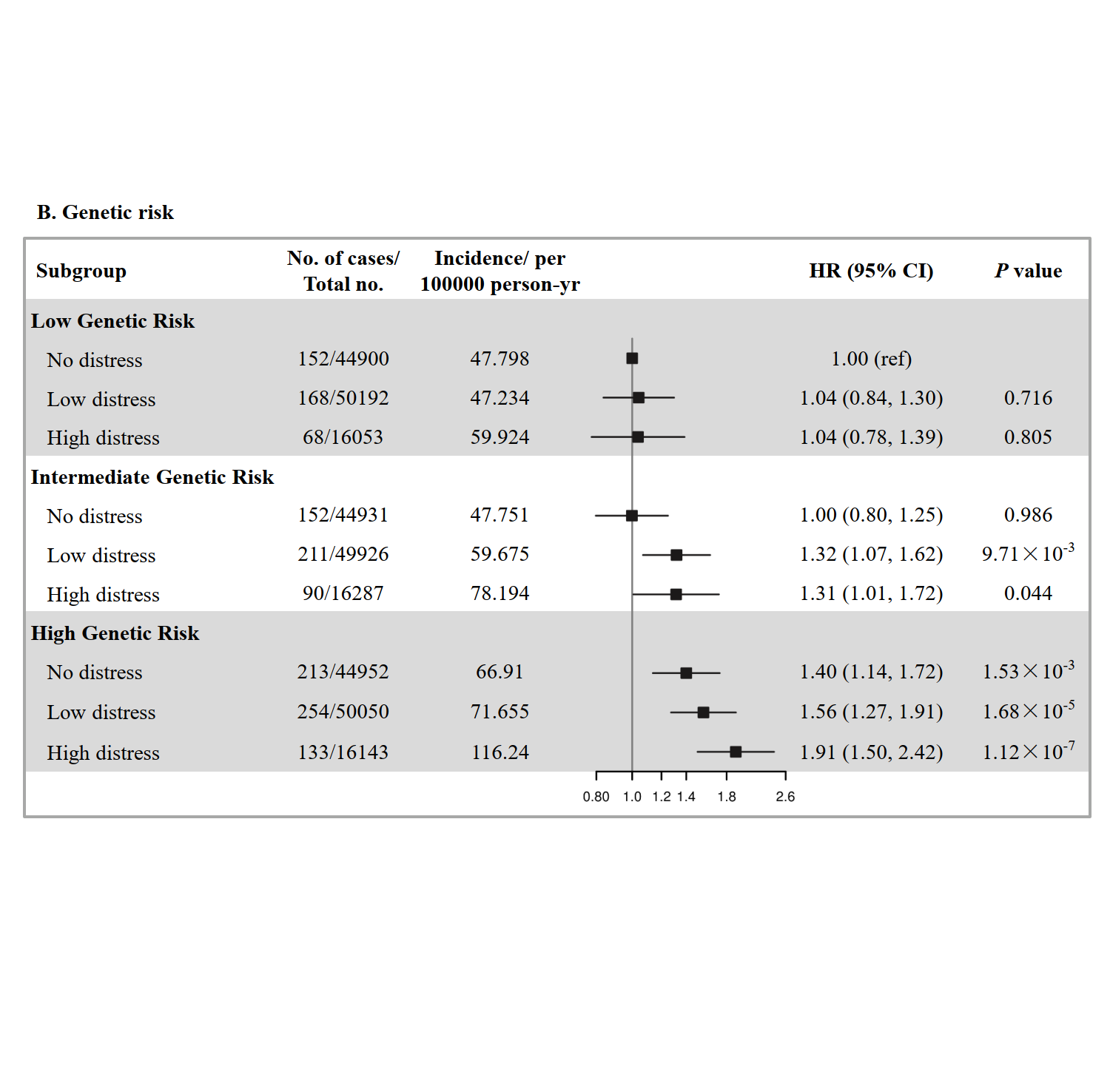


**Supplementary Figure 4. Sensitivity analyses of incident lung cancer risk according to (A) smoking, (B) genetic risk and psychological distress categories in the UK Biobank**. The smoking levels were defined as none (never smoker), light (PY <20) and heavy (PY ≥20). The overall genetic risk of white British individuals was defined as low (lowest tertile), intermediate (second tertile) and high (highest tertile). The psychological distress was defined as none (quartile 1), low (quartiles 2-3) and high (quartile 4).

For the smoking, the hazard ratios were estimated using Cox proportional-hazard models with adjustment for age at recruitment, sex, ethnic background, education, Townsend deprivation index, family history of lung cancer, healthy diet score, BMI, and physical activity. For the PRS, another adjusted for smoking status, pack-years of smoking, the first ten principal components of ancestry and genotyping batch.
